# 

**Published:** 1911-01-28

**Authors:** 


					WORKS BY
Sir FREDERICK TREVES, Bt.,
G.C.V.O., C.B., LL.D., &c.,
Serjeant-Surgeon to H.M. the King, Surgeon in Ordinary to
H.M. Queen Alexandra.
Author of " Tale of a Field Hospital."
With 72 Illustrations from Photographs by the Author,
and a Map.
Second Impression. Small royal 8vo. 9/- net.
Daily Telegraph.?" ' Uganda for a Holiday ' should prove
to be one of the more popular ot' the books of the season
... a work which will afford all readers who delight in
brilliant travel talk a very delectable treat."
Public Opinion.?" Sir Frederick Treves,the famous surgeon,
is making a second reputation as a brilliant writer of books
of travel ... an extraordinarily picturesque writer. He has
much imagination and insight, and is full of interest in the
world."
NEW AND CHEAPER EDITION.
With Illustrations and Maps. Demy 8vo. 6/- net.
Truth.?" Nowhere have I read such vivid pictures at once
of the West Indian Islands as they are to-day, and of th
striking and even startling scenes of which they have been
the theatre in the past."
London:
SMITH, ELDER & CO., 15 Waterloo Place, S.W.
Just Issued. Ninth Edition, Rewritten and Enlarged, 9s. net.
PHARMACY, MATERIA MEDIGA & THERAPEUTICS*
Being a Commentary on the B.P. and its Drugs, with their Preparations,
and on all the new Non-official Remedies in use, with Chapters on the
different processes used in Dispensing, Prescription-writing, Latin
Glossary, &c. By Sir W. WHITLA, M.D., LL.D., Professor, Materia
Medica, Queen's University, Belfast, and Senior Physician, Royal Victoria.
Hospital.
By the same Author. New Work. 1,900 pages. Crown 8vo. 25s. net.
PRACTICE OF MEDICINE.
Containing in two Handy Volumes a complete and independent Article on
the Etiology, Symptoms, and Diagnosis of every Medical Disease; arranged,
in Dictionary Form for Practitioners and Students..
London: BAnxifeRE, Tindall & Cox, 8 Henrietta Street, W.O.,
BOOKS RECEIVED.
S. W. Partridge and Co.
" The Probationer." By A. M. Irving.
J. Nisbet and Co.
" Forbidden Fruit to Young Men." By Lieut. Churchill.
Hazell, Watson, and Viney.
" Smith's Physician's and Surgeon's Visiting List."
J. W. Arro'wsmitii.
" Memories of Eighty Years." By J. Beddoe.
Reigate Press,
" The Church of the Departed." By II. Cashmore.
Henry J. Glaisher.
" Non-Surgical Treatment of Duodenal Ulcer." By
George Herschell.
D. Appleton and Co.
" A Manual of Nursing." By Miss M. Donahoe.
C. S. Welles.
" The Millennium." By C. M. Welles.
Churchill and Liverpool Booksellers.
" The Dawn of the Health Age." By Benjamin Moore.
Pears, Ltd.
" Pears' Shilling Encyclopedia."
Whitaker and Co.
" Whitaker's Peerage, Baronetage, Knightage, and Com-
panionate."
" Whitaker's Almanack."
J. Murray.
" Induced Cell Reproduction and Cancer." By II. C. Ross.
P. Blaiciston, Son & Co.
"Care of the Patient." By Hawes.
T. Fisher Unwin.
" Conquest of Consumption." By A. Latham and C. II.
Garland.
Marshall Bros., Ltd.
" Lepers." By J. Jackson.
Ciiatto and Windus.
" Fry's London Charities.". By J. Lane.
T. C. and E. C. Jack.
" The Bacillus of Long Life." By L. M. Douglas.
Henhy Frowde and Hodder and Stoughton. .
"Handbook of the Surgery of the Kidneys." By W. B.
Clark.
" Text Book of Massage. By L. L. Despard.
" Diseases of the Spinal Cord." By R. T. Williamson.
Bale and Danielson, Ltd.
" Modern Surgical Technique." By C. Y. Pearson.
J. Willing, Jun., Ltd.
" Willing's Press Guide, 1911."
Obituary.
Walter Tyrrell, of London, has died at Malvern
Wells, aged seventy-nine. Mr. Tyrrell became an L.S.A.
in 1853, and an M.R.C.S. in 1854.
Mr. Samuel Parsons-Smith has died at Croydon at the
age of sixty-eight. Mr. Parsons-Smith studied in Dublin,
where he gained the conjoint diploma of the Irish Royal
Colleges.
Miss Harriett Amelia Rachel Apps, of the C.M.S.
Hospital, has died at Nablus, Palestine, at the age of
thirty. Miss Apps studied at the London School of Medi-
cine for Women, and at Durham, where she graduated
M.B., B.S. in 1909.
The death is announced at Chigwell, Essex, of Mr.
Henry Taylor Briekwell, aged sixty-four. He studied at
the London Hospital, took his L.S.A. in 1885, and his
M.R.C.S. in 1884. He was medical officer and public vac-
cinator to the Chigwell district of Epping Union.
The death is announced of Mr. Samuel Parsons-Smith,
formerly Brigade-Surgeon 1st London Volunteer Infantry
Brigade (Royal Fusiliers). He qualified in 1870 in Ire-
land, and was L.M. of the Rotunda Hospital, Dublin, at
which city he had been resident medical officer of the
Mercers' Hospital.
The death is reported at Apsley Guise. Beds., of Mr.
Henry Veasev, F.R.C.S., at the age of ninety-two. Mr.
Yeasey, who was a Guy's man, became a member of the
R.C.S. so long ago as 1840, in which year also he gained
the L.S.A. It was not till twenty-two years later, in
1862, that he obtained his Fellowship.
THE MEDICAL SCHOOLS.
The following are the arrangements for next week at the
Medical Graduates' College and Polyclinic,, 22 Chenies
Street, W.C. : Monday, January 30, at 4 p.m., Dr. J.
Graham Little. Clinique (Skin) ; at 5.15 p.m., Dr. Percy
Kidd, "Some Affections of the Mediastinum"; Tues-
day, at 4 p.m., Dr. James Taylor, Clinque (Med.) ; at
5.15 p.m., Dr. W. H. Stoddart, "On Nervous Exhaus-
tion"; Wednesday, February 1, at 4 p.m., Mr. C. A. R.
Nitch, Clinique (Surg.); at 5.15 p.m., Mr. Lockhart Mum-
mery, " Piles : Their Pathology and Treatment" ; Thurs-
day, at 4 p.m., Mr. E. Laming Evans, Clinique (Surg.) ;.
at 5.15 p.m., Dr. J. E. Squire, "Bronchitis"; at 4 p.m.,
Mr. Herbert Tilley, Clinique (Ear, Nose, and Throat).
The following are the arrangements for next week at
the West London Postgraduate College : January 30 and
February 2, at 10 a.m., Demonstration by Surgical Regis-
trar; February 1, at 10 a.m., Gynaecological Demonstra-
tion; January 31, at 11.30 a.m., Demonstration of Minor
Operations; January 30, at 12 noon, Pathological Demon-
stration; February 2, at 12.15 p.m., Lecture, Dr. Grainger
Stewart, Practical Medicine. Lectures at 5 p.m. :
January 30, Dr. Beddard, Practical Medicine, Lecture 3;
January 31, Dr. Robert Jones, " Preventible Insanities";
February 1, Mr. Lloyd Williams, "Extraction of Teeth;
When and How to Extract"; February 2, Mr. Baldwin,
Practical Surgery, Lecture 3; February 3, Dr. Bernstein,
Clinical Pathology,
Gifts and Bequests.
Mr. James Wilcock, of Blackburn, chairman of the
Refuge Assurance Company, Ltd., who was sixty-five last
week, has given ?1,000 to the Blackburn Infirmary, with
sums to other charities, making a total of ?4,600, as a
thank-offering. Five years ago Mr. Wilcock made similar
gifts.
The Queen has sent a cheque for her annual subscrip-
tion to the Royal Hospital for Incurables, Putney Heath,
of which her Majesty is patron.
Mr. William Henin Christie, of St. Leonards-on-Sea,
has bequeathed the residue of his estate to the Hastings,
St. Leonards, and East Sussex Hospital. The amount
available for the hospital would appear to be over ?11,000.
Mr. Richard Phipps, of Buckenhill, Bromyard, Here-
ford, formerly partner in Messrs. P. and R. Phipps and
Co. (now P. Phipps and Co., Ltd.), brewers and wine
merchants, who left estate valued at ?118,417 gross, has
bequeathed ?1,000 to the Northampton General Infirmary
and ?500 to the Royal Victoria Dispensary, Northampton.
Miss Mary Elizabeth Wemyss, of Gloucester, has be-
queathed ?300 to the Anti-Vivisection Society.
Among the latest contributions to King Edward's Hos-
pital Fund for London is the sum of ?25, the annual sub-
scription of Queen Alexandra.
Mr. Philip Meyer, of Camden Park, Tunbridge Wells,
retired brewer, who left estate valued at ?90,342 O.s. lOrf.
gross, has made the following bequests, amounting to
?11,500 : ?1,000 to the Addenbrooke Hospital, Cambridge ;
and ?500 each to the following institutions : Tun-
bridge Wells General Hospital, the Tunbridge Wells Eye
and Ear Hospital, the Tunbridge Wells Homoeopathic
Hospital and Dispensary, the Surgical Aid Society, St.
John's Hospital for Diseases of the Skin, Leicester Square,
the National Hospital for Diseases of the Heart, Soho
Square, W., the West-End Hospital for Paralysis and
Epilepsy and Diseases of the Nervous System, Welbeck
Street, W., the Central London Throat, Nose, and Ear
Hospital, Gray's Inn Road, W.C., and to the British Home
and Hospital for Incurables, Streatham, and the Royal
Albert Orphan Asylum.
Miss Eliza Charlotte Colley, of Huntly House,
Broomhall Pai'k, Sheffield, has bequeathed ?50 to the
Jessup Hospital for Women, Sheffield.
?546 THE HOSPITAL January 28, 1911.
BUILDING WANTS CONTEMPLATED AND
IN PROGRESS.
Arbroath.?The infirmary managers have again been in
.consultation with Dr. Mackintosh, M.V.O., of Glasgow,
the eminent authority on hospital construction, regarding
the proposed complete reconstruction of the institution
-buildings. There is a sum of about ?5,000 available, and
further handsome promises haVe been made.
Bootle.?The northern wing of the Borough Hospital,
Bootle, is about to be reconstructed.
Bradford.?Designs are invited by the Board of
Management of the Bradford Royal Infirmary for the
rebuilding of a new institution in Duckworth Lane. The
.conditions of the competition and all particulars can be
obtained from the Secretary-Superintendent upon pay-
ment of a deposit of three guineas, which will be returned
on the usual terms. Mr. Keith Young, F.R.I.B.A., has
been appointed adjudicator.
Bristol.?The proposed extension scheme for the
General Infirmary here has been helped immensely by
Lord Winterstoke's gift of ?5,000. We understand that
the name of an architect has not yet been decided on.
Colchester.?The plans, which have been prepared by
the architect Mr. Crusall, for giving thirty additional
beds to the infirmary at Colchester have been discussed
by the guardians. The question of further increasing the
accommodation was also raised.
Fareham.?The question of a new isolation hospital at
Fareham has been placed in the hands of a committee
?appointed by the Board of Guardians.
Inverness.?The advisability of providing a tuberculin
dispensary in the town of Inverness is now being con-
sidered by the Public Health Committee.
London.?The new extension at the Queen Square
Italian Hospital, of which Mr. J. D. Slater was the
architect, was formally opened last week. The new
?building contains a laundry and mortuary post-mortem
room. The out-patients' pharmacy and ophthalmic de-
partments are placed on the ground level, a new isolation
ward and nurses' quarters are on the first floor, and the
second provides operating theatre with sterilising and
ansesthetising rooms and surgeons' quarters, while the third
is given up to servants' accommodation.
Romford.?At a public meeting, presided over by J. R.
Holliday, Esq., a committee was appointed to discuss the
enlargement of the Victoria Cottage Hospital.
St. Albans and Harpenden Councils have accepted
a tender for the erection of an isolation hospital at Jolly
Mead, St. Albans. It is proposed to use the building
for diphtheria cases.
St. Austell.?The St. Austell Cottage Hospital Com-
mittee is inviting designs from architects practising in
Cornwall for the new buildings that have been decided
on. They are to have accommodation for ten beds.
Wimbledon.?Plans have been prepared by Mr. T. G.
Jackson, R.A., for North Wimbledon Cottage Hospital.
The Wimbledon Hospital in Kingston Road is to be re-
built. The first portion will consist of administration
block and two wings, one of which will contain the King
Edward VII. ward.
THE WEEK'S NOVELTIES.
THE ROYAL DOULTON POTTERIES COMPANY,
the work of which is so well known to and appreciated by
hospital men, has been appointed, by Royal Warrant,
Potters to His Majesty King George V.
The rubber boom being nearly over, there is a general
cheapening of rubber goods all along the line, and institu-
tional appliances, rubber gloves, and bandages share in this
lowering of price. Messrs. H. Statham & Co., who are the
well-known makers of one of the finest qualities of
PATENT POROUS ELASTIC BANDAGES, intimate
that they have considerably reduced the prices of their
goods. Statham's bandages need no recommendation to
those that have once tried them. Those who are as yet un-
acquainted with their excellent points should lose no time
in writing for a sample and price-list. The firm's address
is 9, 10, and 11 Corporation Street, Manchester.
Anyone who wishes to combine in England the benefits of
the open-air treatment with protection from the cold winds
must rely upon a properly constructed revolving shelter.
MESSRS. BOULTON AND PAUL, of Norwich, have
made such a shelter their specialty. Number 450, 8 feet
by 6, with awning and spring Toller, can be obtained from
them, carriage paid, for ?20. There are also smaller sizes.
The popularity in winter of a tonic which has also
accepted claims as a protector against influenza is natural
enough. SCOTT'S EMULSION can, moreover, be tested
by any medical practitioner who cares to write to Scott and
Bowne, Ltd., 10 and 11 Stonecutter Street, Ludgate Circus,
E.C.
The Marchioness of Tweeddale gave a "Bridge"
tournament last Tuesday at her house in Hill Street in aid
of the Royal Maternity and Simpson Memorial Hospital
in Edinburgh.
Tie FDTORE of the HOSPITALS.
Medical Relief for All according to means.
A Scheme of Co-operation between the PATIENTS, the
HOSPITALS, and the STATE.
By Sir HENRY BURDETT, K.C.B., K.C.V.O.
Price 2d.
THE SCIENTIFIC PRESS, LTD.,
28 & 29 Southampton Street, Strand, London, W.C.
And at all Bookstalls.
GENERAL PRACTITIONERS'
CONTRIBUTIONS.
The Hospital devotes a special page to General
Practitioners' contributions. We therefore invite
from practitioners contributions based upon their
experience in the management of cases, and in the
treatment and diagnosis of disease; especially shall
we be prepared to welcome articles dealing, practi-
cally, with treatment, and with the use and value
of new remedies and methods.
No article should exceed 1,100 words, and, if
accepted, one guinea for a contribution of this
length will be paid to the writer after publication.
Each communication should be accompanied by a
stamped directed envelope fdr the return of the MS.
if found unsuitable.
Contributions should be written, or preferably
typed, on one side of the paper only, and all articles
sent in are accepted upon the distinct understanding
that they are forwarded to The Hospital only.
Suggestions Invited.
The Editor will welcome suggestions for the estab-
lishment of any new section in The Hospital, and
will be glad to supply information on any subject of
interest or importance to members of the profession
in any part of the world.
The Scientific Press Publications
INSTITUTIONAL WORKS.
HOSPITALS AND ASYLUMS OF THE WORLD.
By SIR HENRY BURDETT, K.C.B., K.C.Y.O.
In Four Volumes with Separate Portfolio containing severa1 hundred Plans.
Vols. I. and II. (ASYLUMS) ... Price ?6 15s. I Vols. III. and IV. (HOSPITALS)
and the Portfolio of Plans ... Price ?9
The Portfolio Of Plans (20 in. X 14 in., drawn to uniform scale), Price j?4 14 6
The Complete Work   ?12 12s.
This magnificent work forms a Complete Survey of the Origin, History, Construction, Administration and Management
of the Hospitals and Asylums of the World, with detailed information concerning the principal Institutions.
Only a few copies remain unsold.
LEDGERS, ACCOUNT BOOKS, STOCK BOOKS of all kinds for large
and small Institutions.
These are supplied already ruled and printed, or in accordance with the special requirements of institution managers.
CHARTS and CHART CASES. ! CASE PAPERS
Temperature Charts? For Day and Night Nurse's Reports- Bound & nnbound.
Fonr-uonTchart?'"! 1 25 I it DISTRICT NURSING ASSOCIATIONS'
Maternity Charts ... j 1,000 ... 23s. Case, Visit, Time, and Loan and Account Books.
INSTITUTIONAL LIBRARY.
THE SCIENTIFIC PRESS will procure literature, professional and otherwise, for the formation of
and addition to Institutional Libraries. It is economy of time and expenditure to utilise one source
of supply.
THE SCIENTIFIC PRESS, LTD.,
28 and 29 Southampton Street, Strand, London, W.C.

				

